# Modelling of Hepatitis B Virus vertical transmission dynamics in Ethiopia: a compartmental modelling approach

**DOI:** 10.1186/s12879-023-08343-4

**Published:** 2023-05-31

**Authors:** Denekew Tenaw Anley, Mequanente Dagnaw, Daniel Gashaneh Belay, Dawit Tefera, Zemenu Tadesse Tessema, Ayenew Molla, Sebwedin Surur Jemal, Edget Abebe Zewde, Melkalem Mamuye Azanaw, Getachew Aragie, Yayehirad Alemu Melsew

**Affiliations:** 1 Department of Public Health, College of Health Sciences, Debre Tabor University, Debre Tabor, Ethiopia; 2 Department of Epidemiology and Biostatistics, Institute of Public Health, College of Medicine and Health Sciences, University of Gondar, Gondar, Ethiopia; 3 Department of Statistics, College of Natural and Computational Science, Mizan Tepi University, Mizan Tepi, Ethiopia; 4 Department of Biomedical Sciences, College of Health Sciences, Debre Tabor University, Debre Tabor, Ethiopia; 5 Department of Padiatrics and Child Health Nursing, Debre Tabor University, Debre Tabor, Ethiopia; 6 Department of Epidemiology and Preventive Medicine, School of Public Health and Preventive Medicine, Monash University, 553 St Kilda Road, Melbourne, VIC 3004 Australia

**Keywords:** Hepatitis B virus, Vertical transmission, Modelling, Ethiopia

## Abstract

**Background:**

Hepatitis B (HB) is a virus which causes a potentially fatal liver infection. It is a DNA virus belonging to the Hepadnaviridae virus family. Africa, after Asia, has the second highest number of chronic HBV carriers and is considered a high-endemic region. Ethiopia is classified as a country with a high prevalence of viral hepatitis and with nations that lack a systematic strategy for viral hepatitis surveillance.

**Methods:**

S-I-C-R deterministic model was developed and the numerical simulations were done in “R” statistical and programming software. Fixed population assumption was considered so as to develop a simple model which could predict the HBV vertical transmission for the next 5 decades.

**Results:**

The model revealed that significant number of populations will be infected and become carrier till the end the next 49 years even though it has decreasing trend. It was predicted that 271,719 people will die of HBV complications if no intervention will be made on its vertical transmission. The sensitivity analysis result showed that the force of infection has the most important parameter in the vertical transmission dynamics of hepatitis B. Provision of hepatitis B immunoglobulin (HBVIG) and vaccines at the time of delivery could decrease the force of infection by more than half and 51,892 lives will be saved if the intervention is offered for 50% of deliveries in Ethiopia.

**Conclusion:**

Despite the fact that the incidence of HBV vertical transmission is substantial, it is expected to decline during the next five decades. However, the situation necessitates immediate attention, since it results in thousands of deaths if no action is taken. Offering HBVIG and vaccinations to the 50% of infants can save many lives and reduces the force of infection by more than a half.

**Supplementary Information:**

The online version contains supplementary material available at 10.1186/s12879-023-08343-4.

## Background

Hepatitis B (HB) is a virus that can cause a potentially fatal liver infection. It is a DNA virus belonging to the Hepadnaviridae virus family [1, 2]. After the virus enters the body, there is a 1.5-to-6-month incubation period (average 4 months) before symptoms appear. Most people have no symptoms or have a minor disease during the acute phase (the first 6 months following infection). Hepatitis B normally advances silently throughout the chronic phase (> 6 months after infection), with no symptoms for the first 10–20 years [3].

Hepatitis B infection can develop chronic illness in certain patients, meaning it lasts longer than six months. Chronic hepatitis B increases the risk of liver failure, cancer, and cirrhosis, a condition in which the liver scars permanently [2]. A safe and effective vaccine that offers 98–100% protection against [1] hepatitis B is available. Preventing hepatitis B infection averts the development of complications including the development of chronic disease and liver cancer [2].

Hepatitis B virus transmission from mother to infant is referred to as vertical transmission. When the mother has acute hepatitis B near delivery, this happens frequently (70 to 100%), whereas it happens less frequently (0–40%) if the mother is an asymptomatic chronic carrier [4]. Hepatitis B surface antigen (HBsAg) testing and hepatitis B immunoglobulin (HBIG) and hepatitis B (HepB) vaccine given to their infants soon after birth are 80–95 percent successful in preventing viral transmission from mother to child [5].

According to the 2013 World Health Organization (WHO) global health impact report on viral hepatitis, chronic liver disease caused by HBV and HCV afflicted more than 240 and 150 million people, respectively. Africa, after Asia, has the second highest number of chronic HBV carriers and is considered a high-endemic region [6, 7].

Ethiopia is classified as a country with a high prevalence of viral hepatitis and with nations that lack a systematic strategy for viral hepatitis surveillance[8, 9]. This indicates that HBV is the neglected disease in Ethiopia despite its endemicity in most regions.

The population of Ethiopia is growing very fast, and this indicates the necessity of a strong and reliable health system that can save people from infections like hepatitis virus infections. According to the United Nations (UN) World Population Prospects 2019 report, the total population of Ethiopia was estimated to be about 110 million. According to the reports of the central statistical agency, Women of Reproductive Age (WRA) account for about 23.4% of the total population [10, 11]. Pregnant women are estimated to be about 4.37% of the WRA. HBV seropositivity among pregnant women is estimated to be about 4.7% [12]. The transmission rate is also very significant, for about 70% of infants of HBV-positive mothers are estimated to be infected by the virus, According to different studies, near to 90% of infected infants are estimated to go to the carrier state with lifetime death of about 20% [13]. These pieces of evidence clearly show that the vertical transmission of HBV is a very critical issue as far as controlling it in the country is concerned. Hence, the aim of this study was to model the vertical transmission dynamics of HBV in Ethiopia. It would provide evidence on the vertical transmission dynamics of HBV in the general community as it is the first of its kind in our setting and also it would provide evidence on the impact of an intervention on the reduction of vertical transmission of HBV in Ethiopia.

## Methods and materials

In this study, no humans are involved. A compartmental modelling approach was employed to see the vertical transmission dynamics of HBV. Both one-way and multi-way sensitivity analyses were done to establish the relationship between parameters and to identify the maximum incidence of HBV infection. For each compartment, the theoretical assumptions and calculations were determined in the following headings.

### Background susceptibility

The newborn of HBV infected mothers are susceptible. We also assumed that the population is fixed, the natural birth rate equals the death rate (μ).

### Exposure/pre-infectious/Latency

After infection, there is a period of latency. The Hepatitis B virus has an average incubation period of about 75 days. Then, it progresses to acute infection. The person with acute infection may recover or become a chronic carrier of the virus [2]. However, we assumed that there is no latency period in the vertical transmission of HBV, for it has rapid progress in infants.

### Infectious

The model assumes that the offspring of the exposed and infected individuals are infected with the disease at birth, giving vertical transmission of the disease.

### Recovery/death/immunity

Patients of HBV, either die of complications of the virus like liver cirrhosis and cancer or they may recover from acute infection with permanent immunity [2].

### The transition rates from “latent”, “acute”, and “carrier” compartments

Different literature have found the values of parameters in the HBV vertical transmission dynamics. The average time the infected individuals spend in the acute period is 3 to 4 months. The rate at which individuals leave the acute class is defined as 3.467 per year. The Proportion of acute infection individuals who become chronic carriers is 0.885. The proportion of perinatal infection is 0.7(0.7–0.9) [14, 15]. The other study conducted in Japan had the following parameter values Susceptible (S) to Latent period (L) λ = 0.035 (0.02897–0.04099), Latent period (L) to Active viral replication (T) γ = 1.112 (0.16735–2.05597), Active viral replication (T) to Carrier (C) δ = 0.048 (0.03079–0.06445), Active viral replication (T) to Recovery (R) τ1 = 0.119 (0.09293–0.14596) and Carrier (C) to Recovery (R) τ1 = 0.014 (0.00383–0.02387) [16].

### Model structure

**Fig. 1:**
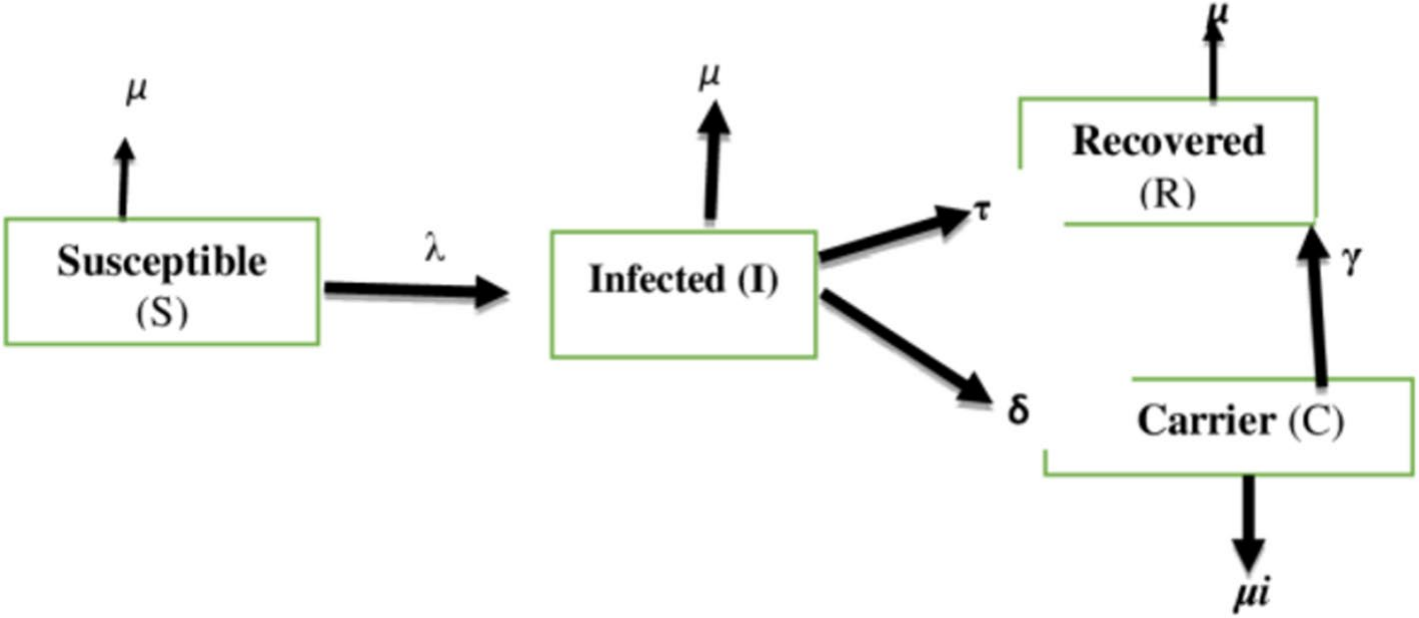
Schematic representation of the vertical epidemiological transmission of HBV infection

This study involved deterministic modelling where the population was partitioned into four components or classes based on the epidemiological state of individuals. The model structure which was selected is based on the natural history of HBV, its nature in the vertical transmission, and general model assumptions to make it simple. The following figure shows the model structure which includes compartment boxes, flow arrows, and parameter symbols (Fig. 1).

### Model description

The above diagram (Fig. 1), shows the model structure of the study. As it is indicated, the population is sub-grouped into 4 compartments. The first compartment is susceptible groups. These groups are prone to infection of HBV by the rate of lamda (λ). The absence of inward arrows towards the susceptible compartment indicates the assumption of a fixed population.

The second compartment is the acute infection groups of the population. These groups may also recover with immunity (the third compartment) at the rate of (τ) or go to a chronic carrier state (the fourth compartment) at the rate of delta (δ). Chronic carriers may die of disease related by the rate of ***µi*** or may recover by the rate of gamma(γ). The parameter symbols are described in the model parameterization part of this study.

### Model equations

#### Ordinary differential equations (ODE)

Deterministic models aim to describe what happens “on average” in a population. These describe the transitions between categories by applying average transition rates. Most of the models in the literature have been deterministic, largely because they are relatively easy to set up and for many purposes knowing what happens on average is sufficient. Deterministic models can be set up using difference or differential equations [17]. In this study, we have used the ODEs to set up the model of the HBV vertical transmission dynamics.

ODEs describe the rate of change in the number of susceptible, acute, carrier, and recovered compartments at time t. The notations are described as follows:

$$\frac{dS\left(t\right)}{d\left(t\right)}= \mathrm{The rate of change in the number of susceptible individuals at time t}.$$

$$\frac{dI\left(t\right)}{d\left(t\right)}= \mathrm{The rate of change in the number of infected individuals at time t}.$$

$$\frac{dC\left(t\right)}{d\left(t\right)}= \mathrm{The rate of change in the number of carriers at time t}.$$

$$\frac{dR\left(t\right)}{d\left(t\right)}= \mathrm{The rate of change in the number of recovered individuals at time t}.$$

$$\uplambda \left(\mathrm{t}\right)=\mathrm{the rate at which susceptible individuals become infected per unit time},\mathrm{ at time t}.$$

Note that; the minus sign in the model equations indicates that individuals move out of the intended compartment, hence it would decrease the number of individuals in the given compartment. For instance; once, individuals are infected they are no more categorized under the susceptible compartment. That is why we have put the minus sign in the force of infection, lamda ($\lambda$) while calculating the rate of change of the number of individuals in the susceptible compartment.

These equations are written as follows:

$$\frac{dS\left(t\right)}{d\left(t\right)}=\boldsymbol{ }-{\uplambda }_{\left(\mathrm{t}\right)}\boldsymbol{ }\times \boldsymbol{ }{S}_{\left(\mathrm{t}\right)}$$

$$\frac{dI\left(t\right)}{d\left(t\right)}=\boldsymbol{ }{\uplambda }_{\left(\mathrm{t}\right)}\boldsymbol{ }\times \boldsymbol{ }{S}_{\left(\mathrm{t}\right)} - {\varvec{\uptau}}(\mathrm{t})\times \mathrm{ I }(\mathrm{t}) - {\varvec{\updelta}} (\mathrm{t})\times \mathrm{ I }(\mathrm{t})$$

$$\frac{dC\left(t\right)}{d\left(t\right)}={{\varvec{\updelta}}}_{\left(\mathrm{t}\right)\boldsymbol{ }}\times \boldsymbol{ }{\mathbf{I}}_{\left(\mathrm{t}\right)}-{{\varvec{\upgamma}}}_{\left(\mathrm{t}\right)} \times \boldsymbol{ }{\mathrm{c}}_{\mathrm{t}}\boldsymbol{ }- {{\mu}}_{\mathrm{i(t)}}\times {\mathrm{c}}_{\mathrm{(t)}}$$

$$\frac{dR\left(t\right)}{d\left(t\right)}=\boldsymbol{ }{{\varvec{\uptau}}}_{(\mathrm{t})}\boldsymbol{ }\times \boldsymbol{ }{\mathbf{I}}_{\left(\mathrm{t}\right)}\boldsymbol{ }+\boldsymbol{ }{{\varvec{\upgamma}}}_{\left(\mathrm{t}\right)} \times \boldsymbol{ }{\mathrm{c}}_{\mathrm{(t)}}$$

### Parameterization

#### Parameter estimation

The following table shows the parameters and their values used in the modelling proces**s** (Table 1).

**Table 1:** Parameter values used in the model simulation

Symbol	Name of the symbol	Parameter	Baseline value (Range)	Source
*µ*	Mu	The rate of natural mortality/death rate from each compartment		
*R*_*o*_		Basic reproductive number	3.7	[18]
***Β***	Beta	transmission rate per infectious person per time	0.8	[18]
*µ*_*i*_	Mu	The rate of mortality from complications of HBV in chronic carriers	0.2(0.15–0.25)	Calculated
λ	Lamda	The rate at which susceptible individuals become infected by HBV	0.05(0.025–0.075)	[16]
**τ**	Tau	The rate at which individuals with acute infection of HBV recovers with permanent immunity	0.119 (0.09293–0.14596)	[16]
δ	Delta	The rate at which individuals with acute infection become chronic carrier of the virus	0.048 (0.03079–0.06445)	[16]
Γ	gamma	The rate at which individuals from chronic carrier stage recover from the virus	0.014 (0.00383–0.02387)	[16]

### Estimating initial populations

N.B: Percentages for the determination of the initial population for each compartments were accessed from studies on the prevalence of HBV among pregnant women and infants in Ethiopia. The baseline population was estimated in the following way;

$$\mathrm{Total population of Ethiopia}=\mathrm{ 110,000,000}.$$

$$\mathrm{Female reproductive age group }[15-49] =\mathrm{ Total population }* 23.4\mathrm{\%}.$$

$$=\mathrm{ 110,000,000 }*23.4\mathrm{\% }= 25,740,000$$

$$\mathrm{Pregnant women }(\mathrm{N}) = 15-49\mathrm{ age women }* 4.37\mathrm{\%}.$$

$$=\mathrm{ 25,740 }*4.37\mathrm{\% }=1,124,838$$

$$\mathrm{HBV}+\mathrm{ pregnant women }=\mathrm{ pregnant women }* 4.7\mathrm{\%}.$$

$$=\mathrm{1,124,838 }*4.37\mathrm{\% }= 52,867$$

$$\mathrm{HBV}+\mathrm{ Infants }[\mathrm{infected }(\mathrm{I})] =\mathrm{ HBV}+\mathrm{ pregnant women }* 70\mathrm{\%}.$$

$$=\mathrm{ 52,867 }*70\mathrm{\% }= 37,007$$

$$\mathrm{Carrier infant }(\mathrm{C}) =\mathrm{ infected infants }* 88.5\mathrm{\%}.$$

$$=\mathrm{ 37,007 }*88.5\mathrm{\% }= 32,751$$

$$\mathrm{Death at life time }(\mathrm{ui}) =\mathrm{ carrier }* 20\mathrm{\%}.$$

$$=\mathrm{ 32,751}*20\mathrm{\% }= 6550$$

$$\mathrm{Recovery }(\mathrm{R}) =\mathrm{ infected }*11.5\mathrm{\% }+\mathrm{ carrier }* 80\mathrm{\%}$$

$$=\mathrm{37,007}*11.5\mathrm{\% }+\mathrm{ 32,751 }*80\mathrm{\% }= 30,457$$

$$\mathrm{Susceptible }(\mathrm{S}) =\mathrm{ All pregnant }(\mathrm{N}) - (\mathrm{infected }(\mathrm{I}) +\mathrm{carrier }(\mathrm{C}) +\mathrm{ recovered }(\mathrm{R})) =1,024,623$$

The model assumptions, model structure, tranisition parametres and initial populations estimated were used in the data simulation process, and whole analysis was done using R software. The R codes established in the modelling process are provided as additional files (Additional File 1).

## Results

**Fig. 2:**
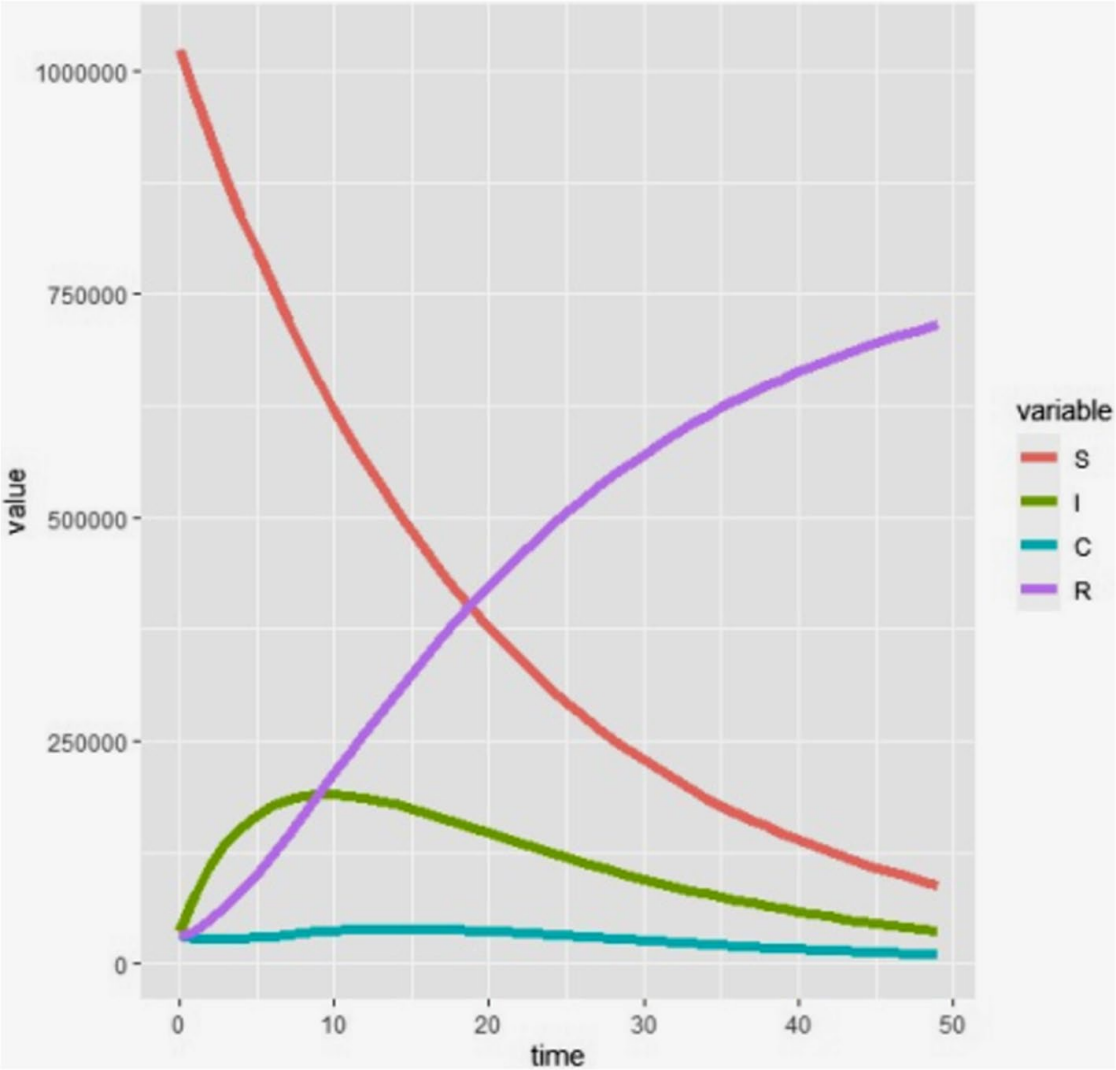
The number of populations in each compartment of the HBV vertical transmission model structure modelled for 49 years, Ethiopia, January 2021

The first output was the one that shows the number of populations in each compartment. It was modelled for 49 years (for the full period of reproductive age). The graph shows the trend of the population in each compartment (Fig. 2).

**Fig. 3:**
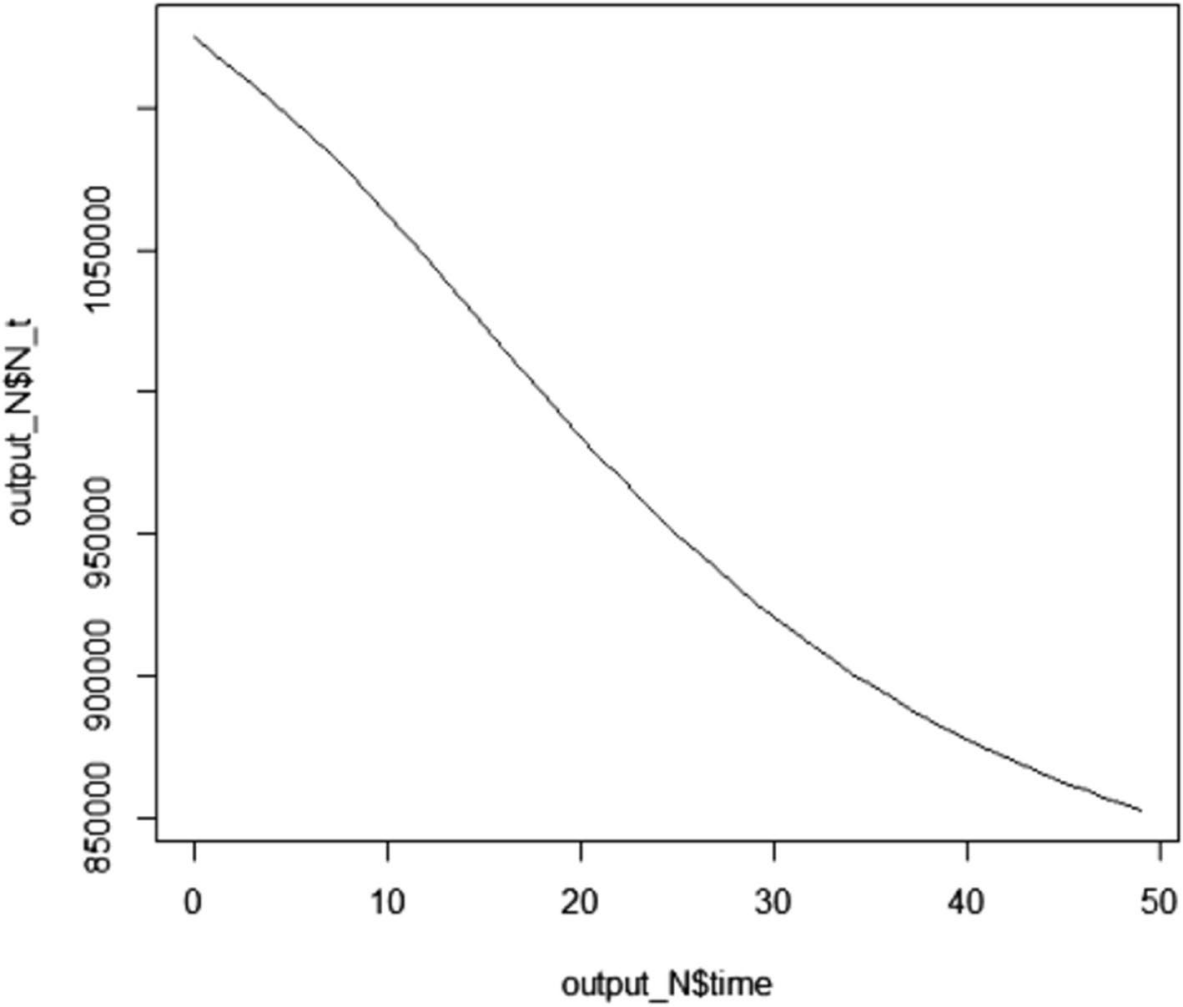
The number of total populations in each year for the next 49 years in HBV vertical transmission modelled for 49 years, Ethiopia, January 2021

At the end of the study period, the overall population (the sum of the population in the four compartments) was found to have decreased by a significant number (Fig. 3).

### Incidence of HBV infection

**Fig. 4:**
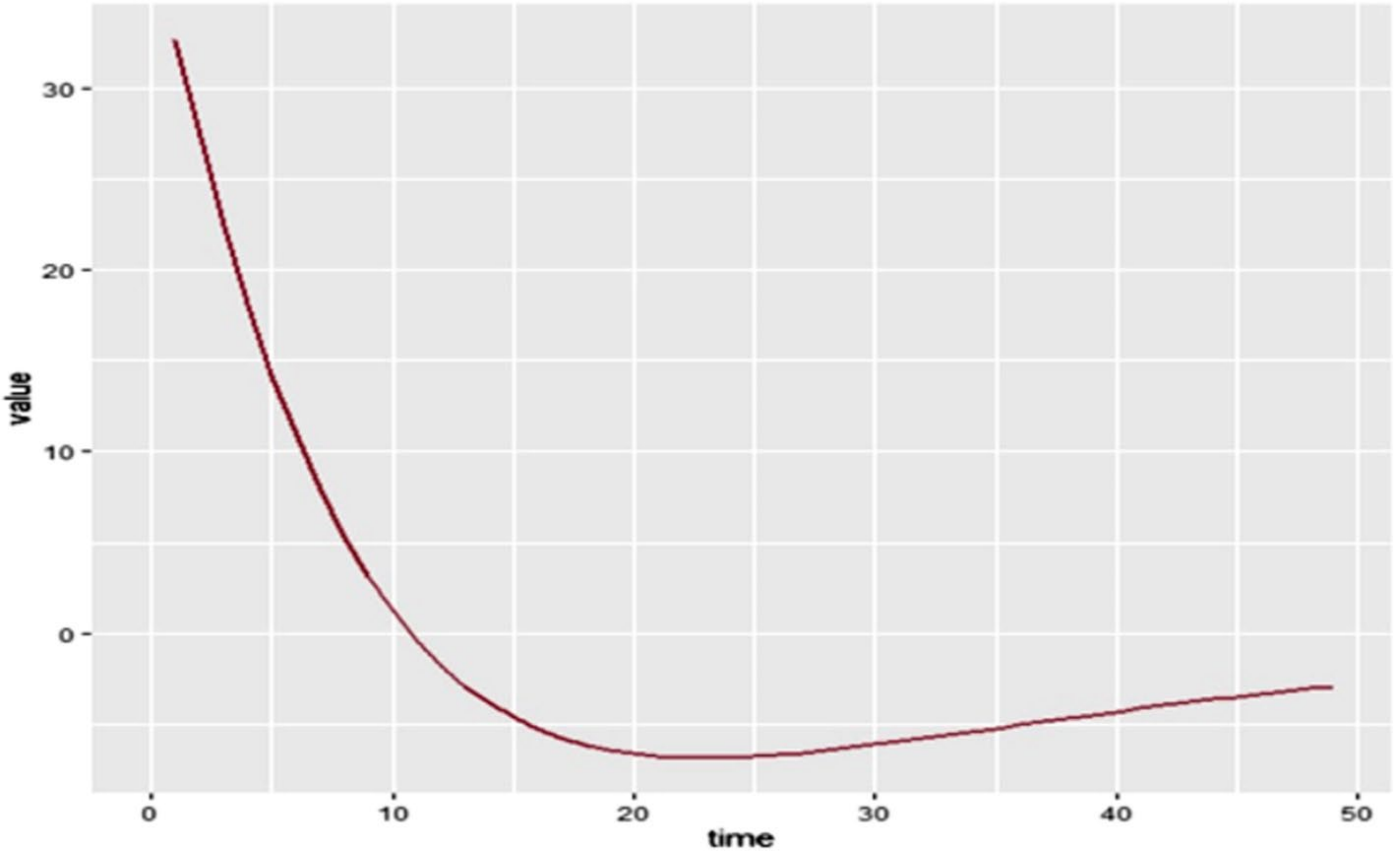
The graphical presentation of incidence of HBV infection in each year in HBV vertical transmission dynamics modelled for 49 years in Ethiopia, January 2021

The incidence in the first year will be 33 per 1000 populations at risk. It will then decrease till the end of the 49 years. The following is the graphical representation of the incidence of HBV infection in each year for the next 49 years. The x-axis of the graph represents years and the y-axis represents the incidence of HBV per 1000 population at risk (Fig. 4).

**Fig. 5:**
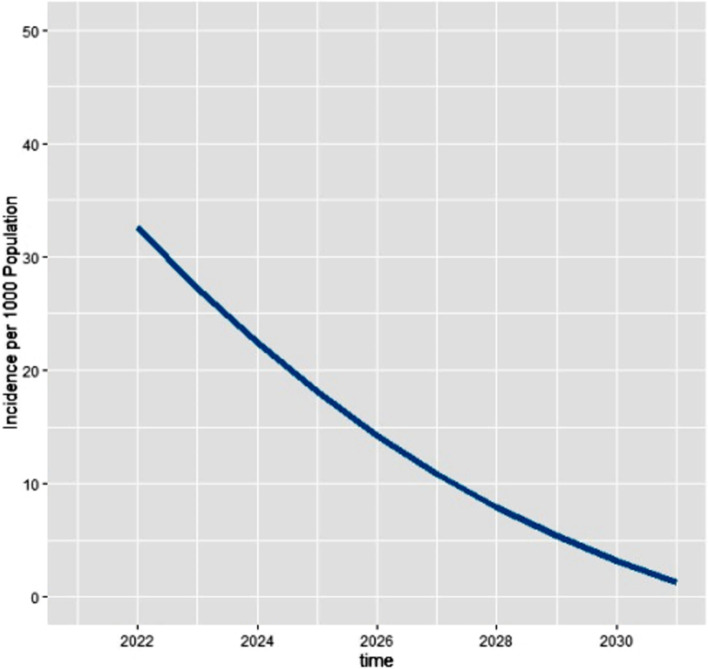
The incidence of HBV infection per 1000 population in HBV vertical transmission dynamics for the first 10 years (2022 to 2031 GC) in Ethiopia, January 2021

The incidence of HBV infection in Vertical transmission was also modelled for the next 10 years, from 2022 to 2031. The incidence decreases as it goes to 2031 (Fig. 5).

### Sensitivity analysis

#### One-way sensitivity analysis

**Fig. 6:**
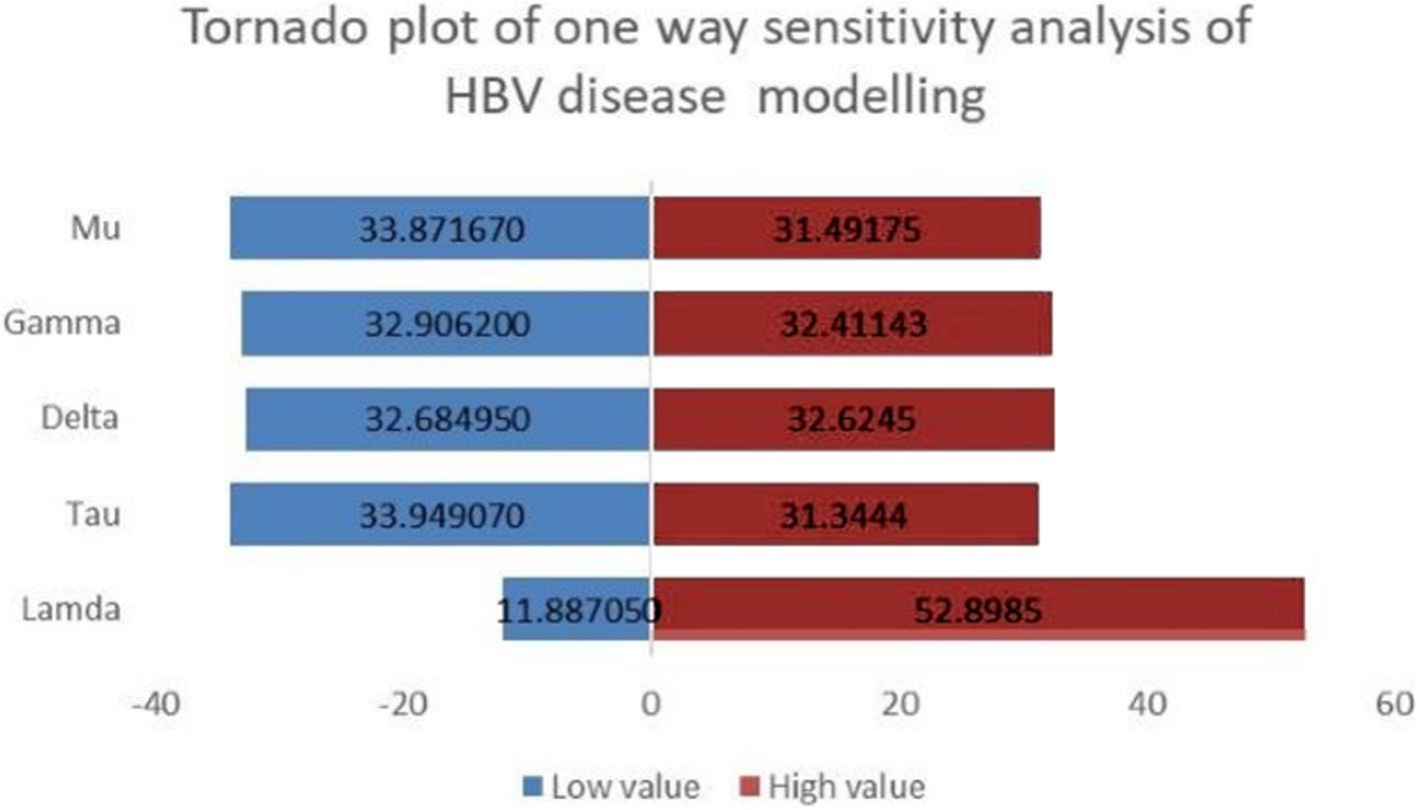
Tornado plot showing the result of uncertainty analysis in HBV vertical transmission dynamics modelled for 49 years

One-way sensitivity analysis was done using the lowest and highest values of the five parameters (lamda, tau, delta, gamma, and mu). The outcome of interest was the maximum incidence of hepatitis B virus infection(Fig. 6).

The low, high, and base values of the maximum incidence were determined using the lowest and highest range parameter values in the one-way sensitivity analysis (Table 2).

**Table 2:** The low, high, and base values of the maximum incidence using the lowest and highest range parameter values in one-way sensitivity analysis of the HBV vertical transmission dynamics modelled for 49 years

	**Low value**	**High value**	**Base value**	**Difference**
**Lamda**	11.88705	52.8985	32.65389	41.01145
**Tau**	33.94907	31.3444	32.65389	-2.60467
**Delta**	32.68495	32.6245	32.65389	-0.06045
**Gamma**	32.9062	32.41143	32.65389	-0.49477
**Mu**	33.87167	31.49175	32.65389	-2.37992

### Multiway sensitivity analysis

**Fig. 7:**
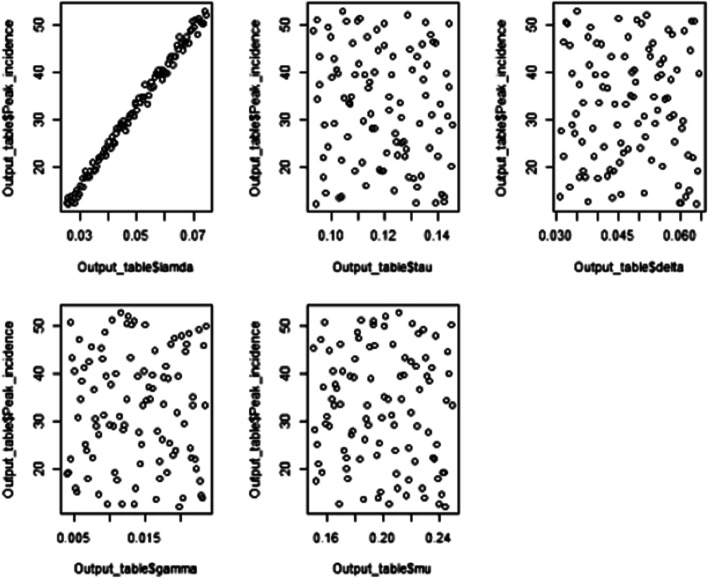
Scatterplot showing the relationship between the peak incidence and different parameters

Multiway sensitivity analysis was performed after one-way sensitivity analysis was done. In this sensitivity analysis, the peak incidence of HBV infection was determined for each parameter sets. Besides, the relationship between the parameters and the maximum incidence of HBV infection was established (Fig. 7).

### Intervention implementation

Hepatitis B immunoglobulin (HBIG) and hepatitis B (HB) vaccine provision can be used as an intervention to reduce the vertical transmission of hepatitis B virus (HBV). We want to plan the intervention, by assuming that it is possible to offer the HBIG and HB vaccine for 50% of deliveries in Ethiopia. The model formulation considering the intervention was done as follows;

Let the intervention to be offered is labeled as: “HBIG_ HB”.

Coverage of HBIG_ HB (C_ HBIG_ HB) = 0.5(50%),

Efficacy of HBIG_ HB (E_ HBIG_ HB) = 0.875(87.5%).lamda_intervention = lamda*(1- (C_ HBIG_ HB* E_ HBIG_ HB))

The value of the force of infection (lamda) after intervention will be:

$$\begin{array}{c}\begin{array}{c}\begin{array}{c}\mathrm{lamda}\_\mathrm{intervention}=\mathrm{lamda}*(1- (\mathrm{C}\_\mathrm{ HBIG}\_\mathrm{ HB}*\mathrm{ E}\_\mathrm{ HBIG}\_\mathrm{ HB}))\\ \mathrm{ lamda}\_\mathrm{intervention}=0.05*(1-(0.5*0.875))\\ \mathrm{ lamda}\_\mathrm{intervention}=0.05*(1-0.4375)\end{array}\\ =0.05*0.5625\end{array}\\ =0.028125\end{array}$$

Therefore, the intervention will reduce the force of infection by 56.25%.

The **ODE** is written as follows:

$$\frac{dS\left(t\right)}{d\left(t\right)}= -{{\mathrm{lamda}}_{\mathrm{intervention}}}_{\left(\mathrm{t}\right)\times } {\mathrm{S}}_{\left(\mathrm{t}\right)}$$

$$\frac{dI\left(t\right)}{d\left(t\right)}= {\mathrm{lamda}\_\mathrm{intervention}}_{\left(t\right)\times \mathrm{s}\left(\mathrm{t}\right)} - {{\varvec{\uptau}}}_{\left(\mathrm{t}\right)\times }{\mathrm{I}}_{\left(\mathrm{t}\right) - }{{\varvec{\updelta}}}_{\left(\mathrm{t}\right)\times }{\mathrm{I}}_{\left(\mathrm{t}\right)}$$

$$\frac{dC\left(t\right)}{d\left(t\right)}= {{\varvec{\updelta}}}_{\left(\mathrm{t}\right)\times }{\mathrm{I}}_{\left(t\right)}-{{\varvec{\upgamma}}}_{\left(\mathrm{t}\right)\times }{\mathrm{C}}_{\left(\mathrm{t}\right)} - {\mu }_{\mathrm{i} \left(\mathrm{t}\right) \times }{\mathrm{c}}_{\left(\mathrm{t}\right)}$$

$$\frac{dR\left(t\right)}{d\left(t\right)}= {{\varvec{\uptau}}}_{\left(\mathrm{t}\right)\times } {\mathrm{I}}_{\left(\mathrm{t}\right) }+ {{\varvec{\upgamma}}}_{\left(\mathrm{t}\right)\times } {\mathrm{C}}_{\left(\mathrm{t}\right)}$$

### The effect of the intervention modelled

**Fig. 8:**
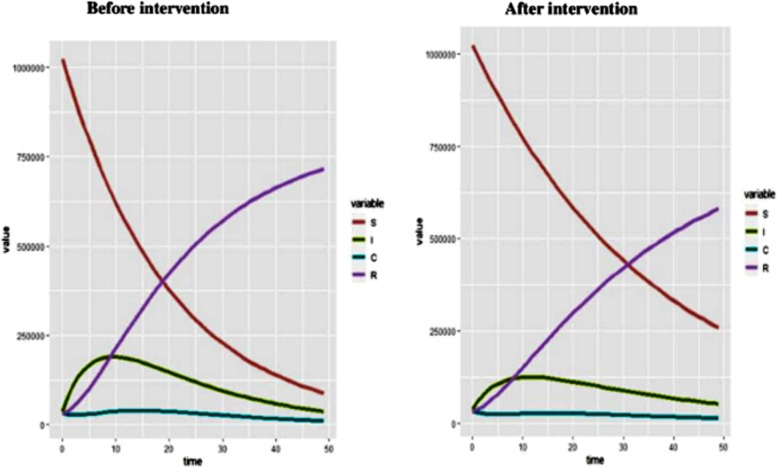
The number of populations in each compartment before and after the intervention in the HBV vertical transmission dynamics modelled for 49 years

The following figure shows the number of populations in each compartment before and after intervention (Fig. 8).

**Fig. 9:**
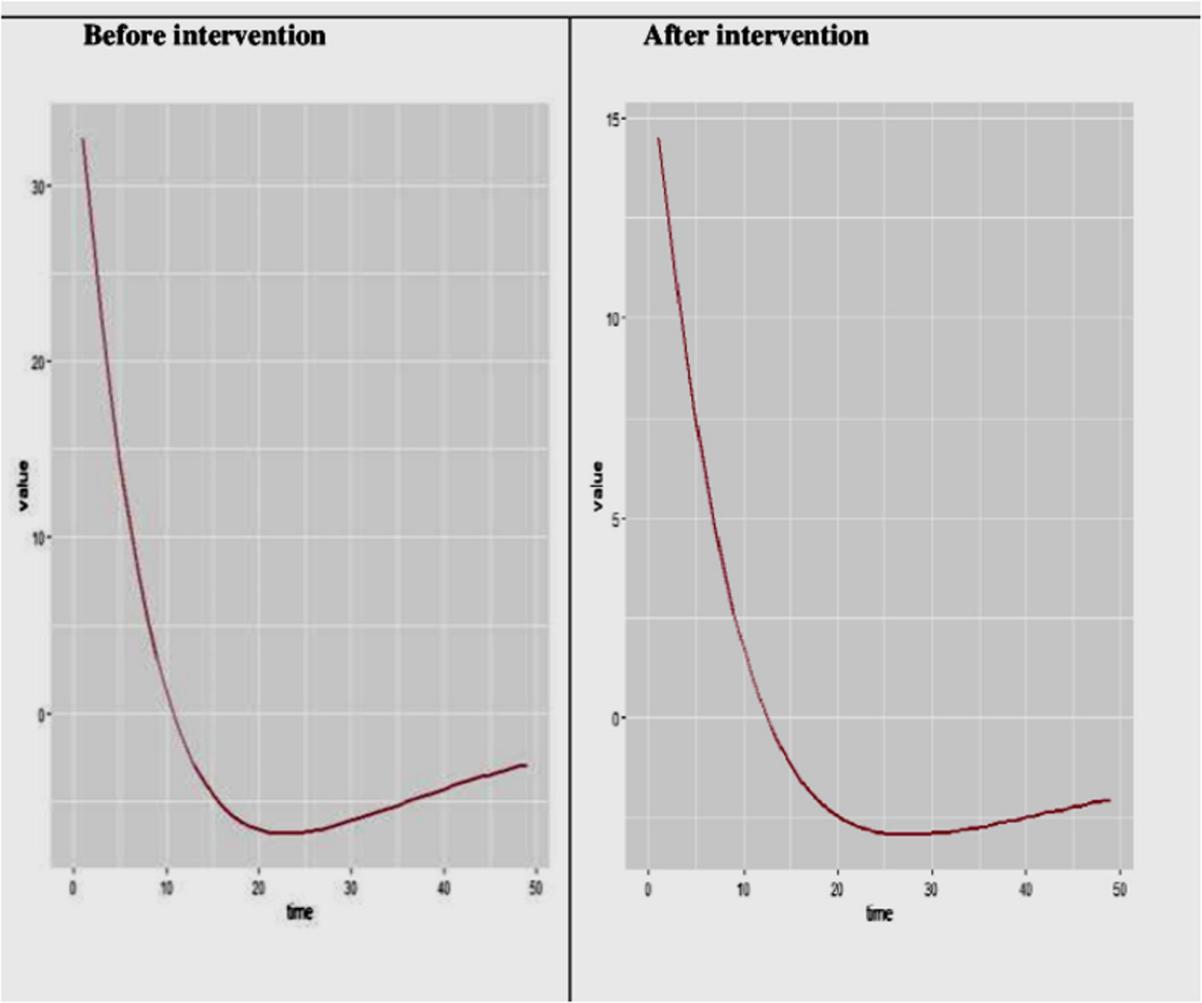
The incidence of HBV infection with intervention in the HBV vertical transmission dynamics modelled for 49 years

The incidence of HBV infection before and after intervention implementation is illustrated in the following graph (Fig. 9).

## Discussion

This study was aimed at modelling HBV vertical transmission dynamics in Ethiopia in which deterministic infectious disease modeling was applied. One-way and multiway sensitivity analysis was done to identify the most important transition parameter in the vertical transmission dynamics of HBV. Then, an intervention that is not being done in Ethiopia currently was implemented in the modelling process to see how it would help save lives and decrease susceptible individuals. The intervention implementation result was presented graphically compared with the condition it would be when there is no intervention. This study is a little bit different from other models developed, for it has included the intervention method and its effect on epidemiological parameters and the incidence of the virus [18–21].

For the sake of model structure identification, information on the natural history of HBV and its vertical transmission was gathered from different literature. According to the natural history of the HBV, the population was partitioned into 4 compartments or classes; namely: Susceptible S(t), Infected I(t), Chronic carrier C(t), and Recovered R(t) compartments making it S-I-C-R model structure. The number of initial populations in each compartment was determined for it was necessary in the modelling process. The estimation of the initial population was done using the current total population of Ethiopia, the proportion of female reproductive age women, the proportion of HBV^+^ pregnant women, the proportion of HBV-infected infants from the total HBV^+^ pregnant mothers, the proportion of carrier infants from total infected infants, lifetime death of carrier infants, total infants in the compartment of recovery (calculated by summing up of recovery from infected and carrier stage) and total susceptible groups were determined.

After the model structure was determined and the initial population was estimated, the ordinary differential equations (ODEs) were written using the transition parameters. These parameters were; mu, lamda, tau and delta, and gamma. The estimation of parameter values was done using different literatures conducted on HBV transmission dynamics across the world.

After the necessary codes were run in “R” statistical software, it was possible to find all the required outputs of the model. The first output was the number of populations in each compartment in the next 49 years. The number of susceptible, Infected, and carrier populations will decrease to 88,434, 37,681, and 10,955 respectively at the end of the study years. The population in the acute infection compartment will increase for 10 years period and will start decreasing then after. The total population in the recovered compartment will increase to 716,048. The population in the carrier compartment will remain almost constant throughout the study period. The number of total populations in each year for the next 49 years was determined with average parameter values and no intervention. At the 49^th^ year, the total population (the sum of the population in the four compartments) will decrease from 1,124,838 to 853,119. This shows that 271,719 people will die of HBV complications if no intervention will be made.

The established model was also needed to determine the incidence of HBV vertical transmission for the next 49 years. The incidence at the first year of the study was found to be 33 per 1000 populations at risk, and it was found to have a decreasing trend over the next 48 years. It was also modelled for 10 years to appreciate the incidence of the disease for the nearest list of years. The incidence of the virus from the beginning of the study period till 2027 will be above 10 per 1000 population at risk, coming to below 10/1000 population at risk then after. However, the identified incidence of the virus in each year of the study is substantial, for the disease’s nature is deadly and highly contagious. Establishing the incidence of the disease for this extended period would be highly beneficial, for it could give insight to concerned bodies to tackle the problem.

The other thing done in the modelling of HBV vertical transmission dynamics was sensitivity analysis. Sensitivity analysis was aimed to identify the most important model parameter in the transmission dynamics of the virus. One-way sensitivity analysis was done and reviled that the base value of the maximum incidence of the HBV infection is 33 per 1000 population when the five parameters are at their average values. The difference of the maximum incidence was found to be the highest (41) when the parameter lamda was at its lowest and highest range. As it is also seen in the Tornado plot of the one-way sensitivity analysis, the parameter lamda (the force of infection) is the most influential parameter in this HBV vertical transmission dynamics.

Multiway sensitivity analysis was also performed for the sake of establishing a clear relationship between the transition parameters and the maximum incidence of HBV infection. For this purpose, the Latin hypercube sampling method, a more efficient way to sample, was used. The model was run for 100 parameter sets or random samples. The correlation of the five parameters with the maximum incidence of the disease was established and presented using a scatter plot. The parameter lamda was found to have a positive linear relationship with the peak incidence of HBV infection. Hence, the two aforementioned sensitivity analysis methods revealed that the force of infection, lamda, is the most important parameter in the HBV vertical transmission dynamics. Furthermore, the finding pointed out where the appropriate intervention should be done to bring the incidence to the level that it will no more be a public health concern in Ethiopia.

Furthermore, this study was able to model an appropriate intervention that can lower the incidence of the HBV infection in the next 49 years. As the sensitivity analysis result pointed out, the intervention has to be targeted at the most influential transition parameter, lamda. Hence, using tests for hepatitis B surface antigen (HBsAg) and providing hepatitis B immunoglobulin (HBIG) as well as hepatitis B (HepB) vaccine to their infants soon after birth is 80–95% effective to prevent mother-to-child transmission of the virus. Currently, the service is not being offered in Ethiopia. As it is indicated, if the service is offered, it will prevent the vertical transmission of the virus by an average of 87.5%. Here, the parameter to be reduced is the force of infection, lamda. It was assumed that the service considered in this model can be offered for 50% of deliveries. The assumption was done considering the expense of the intervention proposed. It was found that the intervention considered could reduce the force of infection by more than half (56.25%).

The result showed that the number of populations in the acute infection compartment is much lower at the beginning of the model and continues to be lower compared to the case with no intervention. The number of susceptible populations will also be much higher at the end of 49 years with the intervention compared to the case with no intervention. The total population in each year is higher after the intervention compared to that where there is no intervention. With the proposed intervention, the total population at the end of the 49^th^ year will be 905,011 from the total population of 1,124,838 at the beginning time, but without intervention, the total population at the end of the 49^th^ year will decrease to 853,119. Regarding the incidence of the infection, the intervention will decrease the incidence of HBV infection by more than 50% (from 33 to 15 per 1000 populations). The role of this intervention in reducing the transmission of HBV is also supported by studies conducted across the world [19, 22, 23].

Generally, this study has brought information about HBV vertical transmission dynamics including the trend of the infection modelled for the full female reproductive period. To the best of our knowledge, it is the first of its kind in Ethiopia, and hence the predictions made for the next 5 decades will be valuable to control the virus. The study has also a strength, for it has included the appropriate intervention and its actual impact on the transmission dynamics of the. However, the study was not without limitations; it would have been better if variables like HIV co-infection and multiple pregnancies were included in the model structure.

## Conclusion

The incidence of HBV vertical transmission will have a decreasing trend for the next 5 decades. The most important parameter in the vertical transmission dynamics of HBV in Ethiopia is the force of infection, and hence interventions shall be planned focusing on this parameter.

About 51,892 lives will be saved if the HBVIG and vaccines are offered for 50% of deliveries in Ethiopia. The intervention can reduce the force of infection by more than half.

## Supplementary Information

**Additional file 1.**

## Data Availability

All data generated or analysed during this study are included in this manuscript and uploaded as supplementary material.

## Abbreviations

DNA: Deoxy Ribo Nucleic acid
GC: Gregorian calendar
HB: Hepatitis B
HBIG: Hepatitis B immunoglobulin
HBsAg: Hepatitis B surface antigen
HBV: Hepatitis B virus
MSEIR: Maternal delivered immunity, Susceptible, Exposure, infection, Recovery
MSIR: Maternal delivered immunity, Susceptible, infection, Recovery
ODE: Ordinary Differential Equations
SICR: Susceptible, Infected, Carrier, Recovery
SEIR: Susceptible, Exposure, infection, Recovery
SI: Susceptible, infection
SIR: Susceptible, infection, Recovery
SIRS: Susceptible, infection, Recovery, Susceptible
